# Association between Dietary Vitamin C Intake and Non-Alcoholic Fatty Liver Disease: A Cross-Sectional Study among Middle-Aged and Older Adults

**DOI:** 10.1371/journal.pone.0147985

**Published:** 2016-01-29

**Authors:** Jie Wei, Guang-hua Lei, Lei Fu, Chao Zeng, Tuo Yang, Shi-fang Peng

**Affiliations:** 1 Health Management Center, Xiangya Hospital, Central South University, Changsha, Hunan Province, China; 2 Department of Infectious Disease, Xiangya Hospital, Central South University, Changsha, Hunan Province, China; 3 Department of Epidemiology and Health Statistics, School of Public Health, Central South University, Changsha, Hunan Province, China; 4 Department of Orthopaedics, Xiangya Hospital, Central South University, Changsha, Hunan Province, China; University of Louisville School of Medicine, UNITED STATES

## Abstract

**Background:**

Non-alcoholic fatty liver disease (NAFLD) has become one of the most prevalent chronic liver disease all over the world. The objective of this study was to evaluate the association between dietary vitamin C intake and NAFLD.

**Method:**

Subjects were diagnosed with NAFLD by abdominal ultrasound examination and the consumption of alcohol was less than 40g/day for men or less than 20g/day for women. Vitamin C intake was classified into four categories according to the quartile distribution in the study population: ≤74.80 mg/day, 74.81–110.15 mg/day, 110.16–146.06 mg/day, and ≥146.07 mg/day. The energy and multi-variable adjusted odds ratio (OR), as well as their corresponding 95% confidence interval (CI), were used to determine the relationship between dietary vitamin C intake and NAFLD through logistic regression.

**Result:**

The present cross-sectional study included 3471 subjects. A significant inverse association between dietary vitamin C intake and NAFLD was observed in the energy-adjusted and the multivariable model. The multivariable adjusted ORs (95%CI) for NAFLD were 0.69 (95%CI: 0.54–0.89), 0.93 (95%CI: 0.72–1.20), and 0.71 (95%CI: 0.53–0.95) in the second, third and fourth dietary vitamin C intake quartiles, respectively, compared with the lowest (first) quartile. The relative odds of NAFLD was decreased by 0.71 times in the fourth quartile of dietary vitamin C intake compared with the lowest quartile. After stratifying data by sex or the status of obesity, the inverse association remained valid in the male population or non-obesity population, but not in the female population or obesity population.

**Conclusion:**

There might be a moderate inverse association between dietary vitamin C intake and NAFLD in middle-aged and older adults, especially for the male population and non-obesity population.

## Introduction

Non-alcoholic fatty liver disease (NAFLD) is the most common cause of liver disease, it can progress to end-stage liver disease and liver failure [1]. NAFLD is characterized by accumulation of hepatic steatosis in patients without excessive alcohol intake [2]. In recent years, the prevalence of NAFLD has increased rapidly both in developed and developing countries. The worldwide prevalence was ranged from 3% to 45% in general population [1,3], and increased in individuals with obesity and diabetes [4,5]. In the United States, around 75 to 100 million people suffer from NAFLD [1]. Meanwhile, the prevalence of NAFLD was between 6.19% and 38.24% In China [6]. NAFLD has become one of the most prevalent chronic liver disease in China which is attracting increasing attention from clinical physicians and medical science researchers.

Previous studies have reported the impact of oxidative stress and inflammation on several chronic diseases, especially on NAFLD, metabolic syndrome, cardiovascular disease, and diabetes mellitus [7–11]. Vitamin C is a powerful antioxidant which is capable of scavenging free radicals, taking part in multiple enzymatic reactions as a reducing agent [12,13]. Moreover, Vitamin C is suggested to be involved in the regulation of both circulating and hepatic lipid homeostasis [14]. Thus, in view of the important function of vitamin C, it probably plays a protective role against NAFLD. However, judging from the current evidences, the association between dietary vitamin C intake and NAFLD remained controversial. Musso et al. [15] suggested a significant lower dietary vitamin C intake in nonalcoholic steatohepatitis (NASH) patients comparing to the healthy controls, but the results were not adjusted by potential confounding factors. Han et al. [16] conducted a cross-sectional study on a sample of only 348 Korean adults, the results of which showed a significant positive association between low vitamin C intake and NAFLD in the male population. In contrast, another small sample (74 NAFLD patients and 27 healthy controls) cross-sectional study suggested that both dietary vitamin C intake and plasma vitamin C concentration were of similar levels between NAFLD patients and healthy controls [17]. In addition, Madan et al. [18] also reported that the plasma vitamin C level of NAFLD patients was not different from healthy controls. However, evidence also weak in view of small sample size (77 individuals).

To our best knowledge, this is the first study which examine the association between dietary vitamin C intake and NAFLD on a large sample with adjustment of potential confounding factors. Further understanding about this association would be helpful for elucidating the pathogenesis of NAFLD, and providing a new insight into the management of NAFLD.

## Method

### Study population

The present study was conducted at the Department of Health Examination Center of Xiangya Hospital between October 2013 and November 2014. The study design has been published previously [19–22]. The protocol of this study was approved by the Ethics Committees on Research of the Xiangya Hospital, Central South University. Written informed consent was obtained from all subjects in this study. Participants were selected according to the following inclusion criteria: 1) aged 40 years or older; 2) availability of abdominal ultrasound examination; 3) completing the semi-quantitative food frequency questionnaire (SFFQ) about the average consumption of foods and drinks over the past 1 year; 4) availability of blood biochemical assessment; 5) availability of all basic characteristics, including age, gender, body mass index (BMI), alcohol drinking status, and smoking status, etc. The following subjects were excluded from the present study: 1) alcoholic fatty liver disease; 2) diabetes patients (fasting glucose ≥ 7.0 mmol/L or currently undergoing drug treatment for blood glucose control); 3) hypertension patients (systolic blood pressure ≥140 mm Hg or diastolic blood pressure ≥90 mm Hg or currently undergoing drug treatment for blood pressure control); 4) severe elevated serum alanine aminotransferase (the normal range is 0–40 U/L, more than 200 U/L is defined as severe elevating). Subjects were diagnosed with NAFLD by abdominal ultrasound examination and the consumption of alcohol was less than 40g/day for men or less than 20g/day for women [23]. 6240 subjects met the inclusion criteria during the study period, then 2499 subjects were excluded based on the presence of diabetes, or hypertension, or alcoholic fatty liver disease, or severe elevated serum alanine aminotransferase. Finally, 3471 individuals were included in the present study.

### Dietary assessment

Dietary intake was evaluated by using a SFFQ which was specially designed for the population in Hunan province, China. The SFFQ has been validated and used in previous studies [19,20]. It contains 63 food items which are popular and commonly consumed in Hunan province. For each food item, participants were asked how frequently (never, once per month, two to three times per month, one to three times per week, four to five times per week, once per day, twice per day, or three times and above per day) they consumed the food in the past year. There are 6 options for the average amount of food consumption in each time: less than 100g, 100-200g, 201-300g, 301-400g, 401g-500g, and more than 500g. Color pictures showing the samples of food with labeled weights were also given to participants to help them make choices more easily. Subjects completed the SFFQ in a self-administered way or interviewed by professional researchers. The Chinese Food Composition Table [24] was used to calculate the individual composition in macronutrients and micronutrients of the included foods. Subjects were also asked if they were taking nutritional supplements or not. The nutritional supplements were described and classified in a crude way (calcium, vitamins, minerals or others).

### Other exposures and health-related behavioral assessment

Registered nurses interviewed all participants during the examination using a standard questionnaire, with the purpose to collect information on demographic characteristics and health-related habits. Participants were asked about their average frequency of physical activity (never, one to two times per week, three to four times per week, five times and above per week) and average duration of physical activity (within half an hour, half an hour to one hour, one to two hours, more than two hours). Education background, smoking and alcohol drinking status were asked face to face. Participants taking any long-term nutritional product, such as calcium, vitamins or mineral supplements, were considered as nutritional supplementation users. All blood samples were drawn after a 12-hour overnight fast and were stored at 4°C until analysis. All blood samples were undertaken routine blood test (including white blood count, red blood count, platelet count, hemoglobin, etc.) and blood chemistry test (including blood glucose, blood lipid, serum electrolytes, trace elements, serum enzyme, uric acid, creatinine, etc.) Laboratory tests were undertaken using a Beckman Coulter AU 5800 (Beckman Coulter Inc., Brea, CA, USA). The weight and height of each subjects were measured respectively to calculate the BMI.

### Statistical analysis

The continuous data (age, activity level, BMI and the dietary intake) were expressed as mean (standard deviation), and the category data were expressed in percentage. Differences in continuous data were evaluated by student’s t test (normally distributed data) or Mann-Whitney U test (non-normally distributed data), while differences in category data were assessed by the χ^2^ test. The vitamin C intake was classified into four categories based on the quartile distribution in the study population: ≤74.80 mg/day, 74.81–110.15 mg/day, 110.16–146.06 mg/day, and ≥146.07 mg/day. The odds ratio (OR) with 95% confidence interval (CI) for the association between vitamin C intake and NAFLD were calculated for each quartile of vitamin C intake, and the lowest quartile was regarded as the reference category. Both energy adjusted model and multivariable adjusted model were adopted for evaluation of the association through logistic regression. The following variables were included in the multivariable adjusted model: age, sex, BMI, cigarette smoking, nutritional supplementary, activity level, education background, dietary energy intake, fiber intake, fat intake, and vitamin E intake. Tests for linear trends were conducted using logistic regression with a median variable of vitamin C intake level in each category. Subgroup analysis were conducted by stratified the data by sex, by the status of menopause in female population, and by the status of obesity(BMI≥28 kg/m^2^, or BMI<28 kg/m^2^). All data analyses were performed using SPSS 17.0 (SPSS Inc., Chicago, IL, USA), and P<0.05 was considered to indicate statistical significance. All tests were two tailed.

## Results

A total of 3741 subjects (1550 males and 1921 females) aged from 40 to 80 years old were included in the present study. The overall prevalence of NAFLD in the study population was 28.8%, which was closed to other similar cross-sectional studies conducted in China [25–28]. The basic characteristics of subjects with or without NAFLD were listed in Table 1. The comparison of the two groups exhibited significant differences in terms of sex ratio, nutritional supplementation, education background, BMI, obesity, menopause, dietary energy intake, fiber intake, fat intake and vitamin E intake.

**Table 1:** Basic characteristics of the study population (n = 3471).

Basic characteristics	NAFLD	Non-NAFLD	P
N (%)	998 (28.8)	2473 (71.2)	-
Age (years)	51.70 (6.76)	51.81 (7.09)	0.955
Sex (%)			<0.001
Male	56.2	40	
Female	43.8	60	-
Cigarette smoking (%)			0.078
Yes	21.0	18.4	-
No	79.0	81.6	-
Nutritional supplementary (%)			0.020
Yes	34.3	38.5	-
No	65.7	61.5	-
Education Background (%)			<0.001
High school or above	52.7	44.5	-
Below high school	47.3	55.5	-
Activity level (h/week)	2.08 (3.27)	2.44 (3.65)	0.057
BMI (kg/m^2^)	26.07 (2.56)	22.69 (2.47)	<0.001
Obesity (BMI≥28 kg/m^2^, %)	21.6	1.9	<0.001
Menopause (% in women)	52.6	43.3	0.001
Dietary energy intake (Kcal/day)	1578.52 (663.41)	1560.31 (699.19)	0.096
Dietary fiber intake (g/day)	18.14 (13.75)	17.35 (14.5)	0.024
Dietary fat intake (g/day)	74.70 (31.14)	72.57 (32.72)	0.004
Dietary vitamin E intake (mg/day)	29.38 (14.16)	28.63 (14.03)	0.030
Dietary vitamin C intake (mg/day)	119.28 (77.17)	123.10 (84.66)	0.516

Continuous data were expressed as mean (SD). NAFLD: Non-alcoholic fatty liver disease, BMI: body mass index.

The results of the energy and multivariable adjusted association between vitamin C intake and NAFLD were shown in Table 2. The energy adjusted OR value suggested an inverse association in the highest quartile of vitamin C intake and NAFLD comparing with the lowest quartile (OR = 0.75, 95%CI: 0.59–0.95, P = 0.016). However, there was no significant linear trend for the association between vitamin C intake and NAFLD (P = 0.063). With adjustment of age, sex, BMI, cigarette smoking, nutritional supplementary, activity level, education background, dietary energy intake, fiber intake, fat intake, and vitamin E intake, the OR showed a decreased prevalence of NAFLD in the second quartile (OR = 0.69, 95%CI: 0.54–0.89, P = 0.003) and the highest quartile of vitamin C intake (OR = 0.71, 95%CI: 0.53–0.95, P = 0.019) in comparison with the lowest quartile. There was no significant linear trend for the association between vitamin C intake and NAFLD (P = 0.098) either. Outcomes of subgroup analysis were listed in Table 3. In the male population, the negative association between vitamin C intake and NAFLD were remained valid in the second quartile (OR = 0.61, 95%CI: 0.43–0.87, P = 0.006) and the highest quartile of vitamin C intake (OR = 0.63, 95%CI: 0.42–0.95, P = 0.029) in comparison with the lowest quartile. But there was no significant association between vitamin C intake and NAFLD in female population. After stratified the data by the status of menopause in female population, same conclusions were drawn. In addition, after stratified the data by the status of obesity, the significant inverse association between dietary vitamin C intake and NAFLD was still existed in non-obesity population (OR = 0.83, 95%CI: 0.55–0.96, P = 0.024 in the highest quartile, OR = 0.75, 95%CI: 0.59–0.96, P = 0.020 in the second quartile), but disappeared in obesity population.

**Table 2:** Energy and multivariable adjusted relationship between dietary vitamin C intake and NAFLD.

	Quartiles of dietary vitamin C intake				P for trend
	Q1 (lowest)	Q2	Q3	Q4 (highest)	
Median vitamin C intake (mg/day)	51.71	92.14	125.53	182.01	-
n	868	868	869	866	
NAFLD (%)	30.1	26.6	31.6	26.7	
Energy adjusted OR (95%CI)	1.00 (reference)	0.81 (0.66, 1.00)	1.01 (0.82, 1.25)	0.75 (0.59, 0.95)	0.063
P value	-	0.052	0.927	0.016	-
Multivariable adjusted OR (95%CI)	1.00 (reference)	0.69 (0.54, 0.89)	0.93 (0.72, 0.20)	0.71 (0.53, 0.95)	0.098
P value	-	0.003	0.933	0.019	-

NAFLD: Non-alcoholic fatty liver disease, OR: odds ratio, 95%CI: 95% confidential interval, multivariable adjusted OR: adjusting by age, sex, BMI, cigarette smoking, nutritional supplementary, activity level, education background, dietary energy intake, fiber intake, fat intake, and vitamin E intake.

**Table 3:** Subgroup analysis of multivariable-adjusted relationship between dietary vitamin C intake and NAFLD.

	Quartiles of dietary vitamin C intake				P for trend
	Q1 (lowest)	Q2	Q3	Q4 (highest)	
Male population					
Multivariable-adjusted OR (95%CI)	1.00 (reference)	0.61 (0.43, 0.87)	0.74 (0.52, 1.07)	0.63 (0.42, 0.95)	0.083
P value	-	0.006	0.112	0.029	
Female population					
Multivariable-adjusted OR (95%CI)	1.00 (reference)	0.79 (0.55, 1.13)	1.20 (0.84, 1.71)	0.85 (0.56, 1.28)	0.807
P value	-	0.200	0.326	0.433	
Premenopause female population					
Multivariable-adjusted OR (95%CI)	1.00 (reference)	0.69 (0.41, 1.14)	1.35 (0.80, 2.27)	0.79 (0.43, 1.42)	0.845
P value	-	0.148	0.266	0.425	
Menopause female population					
Multivariable-adjusted OR (95%CI)	1.00 (reference)	0.93 (0.56, 1.55)	1.04 (0.64, 1.71)	0.91 (0.51, 1.63)	0.795
P value	-	0.790	0.870	0.758	
Non-obesity population (BMI<28 kg/m^2^)					
Multivariable-adjusted OR (95%CI)	1.00 (reference)	0.75 (0.59, 0.96)	0.92 (0.72, 1.17)	0.83 (0.55, 0.96)	0.081
P value	-	0.020	0.486	0.024	
Obesity population (BMI≥28 kg/m^2^)					
Multivariable-adjusted OR (95%CI)	1.00 (reference)	1.15 (0.45, 2.92)	1.55 (0.58, 4.17)	1.20 (0.39, 3.67)	0.699
P value	-	0.777	0.386	0.754	

NAFLD: Non-alcoholic fatty liver disease, OR: odds ratio, 95%CI: 95% confidential interval, multivariable adjusted OR: adjusting by age, BMI (excluded from the obesity and non-obesity subgroups), sex (excluded from all sex subgroups), cigarette smoking, nutritional supplementary, activity level, education background, dietary energy intake, fiber intake, fat intake, and vitamin E intake.

## Discussion

The present cross-sectional study examined the association between dietary vitamin C intake and NAFLD on a large sample comprising 3471 middle-aged and older adults, which suggested that dietary vitamin C intake might moderately inversely associated with NAFLD, especially for the male population and non-obesity population. The inverse association might not be dose-dependent or lineal.

Oxidative stress is suggested to be a key factor in the development and progression of NAFLD [7]. The increased generation of reactive oxygen species (ROS) can lead to lipid peroxidation, which can result in inflammation and fibrogenesis by activating the stellate cells [29]. ROS can also restrain hepatocytes to secret the very low density lipoprotein, and lead to liver fat accumulation. Moreover, oxidative stress can accelerate insulin resistance and inflammation in hepatocyte, which are also critical mechanisms in hepatic dyslipidemia [30]. Vitamin C plays an important role as antioxidant in human health. It takes part in multiple oxidation-reduction reaction as powerful reducing agent, and most of these reactions help regulating both circulation and hepatic lipid homeostasis Vitamin C can decrease mitochondrial ROS formation and improve the activity of manganese superoxide dismutase (SOD) and glutathione peroxidase (GPx) in isolated rat liver mitochondria [31]. It is also suggested to be inversely associated with C-reactive protein (CRP) and myeloperoxidase, which are inflammatory markers [32,33]. Moreover, Vitamin C may impact the regulation of adiponectin, which is suggested to be able to decrease hepatic lipid accumulation, systemic insulin resistance and inflammation, and produce protective effects against NAFLD [34,35]. Vitamin C also acts as a co-factor in the conversion of cholesterol to 7α-hydroxycholesterol, which is the rate-limiting step in bile acid formation. So the deficiency of vitamin C can result in decreased excretion of cholesterol in animals [36]. Several animal studies suggested that vitamin C treatment could effectively relief the hepatic oxidative stress. Nambisan et al. [37] found out that vitamin C deficiency accelerated the dyslipidemia and hepatic consequences, while increased vitamin C intake could reduce the severity of both dyslipidemia and hepatic lipid accumulation in guinea pigs. Rezazadeh et al. [38] proved that vitamin C supplementation significantly decreased hepatic markers of oxidative stress, hepatocellular ballooning and inflammation, while SOD and catalase were increased in rats model.

Although, several randomized controlled trials investigated the treatment effect of vitamin C supplementation on NAFLD, the results were inconclusive. Harrison et al. evaluated the efficacy of the combination vitamin E and vitamin C in reducing histologic inflammation and fibrosis in NASH patients. They concluded that the vitamins treatment achieved an improvement of hepatic fibrosis [39]. Foster et al. suggested that atorvastatin 20 mg combined with vitamins C and E is effective in reducing the odds of having hepatic steatosis by 71% in healthy individuals with NAFLD at baseline after 4 years of active therapy [40]. In contrast, another two trials suggested that the combination of vitamin C and vitamin E supplementation did not achieve a better treatment effect than lifestyle intervention in children with NAFLD [41,42]. However, none of the aforementioned trials assessed the independent effect of vitamin C supplementation, and there were significant differences between children and adults in terms of liver physiology, development and the features of NAFLD, which can result in different outcomes [43]. Our study supported a significant inverse association between dietary vitamin C intake and NAFLD among middle-aged and older adults, especially for the male population and non-obesity population. The results were consistent with another similar research conducted in Asia [16]. The sex-based difference might be caused by the protective effects of estrogen against NAFLD in female [44], which might weaken the association between dietary vitamin C intake and NAFLD in the female population. Similarly, NAFLD is strongly associated with obesity [14,45]. Obesity can lead to low-grade systemic inflammation and redox imbalance with increased oxidative stress [46,47]. So, we speculated that the protective effects from vitamin C intake has been countervailed by obesity. Further studies are required to elaborate such finding.

The present study had several strengths. This is the first study examined the association between dietary vitamin C intake with NAFLD on a large sample with adjustment of a considerable number of potential confounding factors, which greatly improved the reliability of the results. This is also the first study examined the aforementioned association in the Chinese population. Different ethnic groups or populations may exhibit different characteristics. However, limitations of the present study should also be admitted. First, this cross-sectional study is unable to explain the causal relationship, so further prospective cohort studies should be undertaken to establish a causal association between dietary vitamin C intake and NAFLD. Second, the plasma vitamin C concentration was not measured for the target population in this study, which will be addressed in our future investigations. According to previous studies, the vitamin C intake was correlated with median vitamin C plasma concentration [17]. And NASH patients had lower plasma vitamin C levels alongside increased plasma malondialdehyde and nitric oxide compared with steatosis patients, which suggested an ability of vitamin C to prevent the progression of NAFLD [48]. However, Madan et al. did not find any differences in vitamin C plasma levels between 29 NAFLD patients and 23 healthy controls [18]. So, the relationship between plasma vitamin C concentration and NAFLD still need more studies to elaborate. Third, we did not calculated the specific vitamin C intake from nutritional supplements. Because the nutritional supplements were very hard to record and calculated (subjects had the nutritional supplementation by different types, different duration and different doses). Future studies which focus on the effects of vitamin C supplementation may be needed.

## Conclusion

In conclusion, there might be a moderate inverse association between dietary vitamin C intake and NAFLD in middle-aged and older adults, especially for the male population and non-obesity population.

## Supporting Information

S1 File STROBE Statement—Checklist of items that should be included in reports of cross-sectional studies. (DOC)

S2 File PLOS ONE Clinical Studies Checklist. (DOCX)

S3 File Ethics approval. (DOCX)
