# Supplementary material for: Association between Dietary Vitamin C Intake and Non-Alcoholic Fatty Liver Disease: A Cross-Sectional Study among Middle-Aged and Older Adults
Source: PLoS One. 2016 Jan 29;11(1):e0147985. doi: 10.1371/journal.pone.0147985 (PMC4732670; doi:10.1371/journal.pone.0147985)
Supplement: S3 File — (DOCX) [file pone.0147985.s003.docx]

Dear editor:

This is the scanning of the original ethics approval from the Ethics Committees on Research of the Xiangya Hospital, Central South University.

This is the English translation for the ethics approval:

Ethics Committees on Research of the Xiangya Hospital, Central South University.

Ethics Approval

NO. 201312459

| Name | Xiangya Hospital Health Management Center study, XYHMCS | | |
| --- | --- | --- | --- |
| Department | Department of Orthopaedics | Responsibility: In charge | Primary investigator: Guang-hua Lei |
| Classification* | 1 2 3 4 **5** | Study period: October 2013 to December 2015 | |
| Study Type | Observational study | | |
| Institution | Xiangya Hospital, Central South University | | |
| Review opinions | Agree | | |
| Main content related to human in this research:  Study participants were recruited when they attended the Health Management Center for regular health examination. They were undergone a dietary assessment with a structured questionnaire, covering demographic information and data on lifestyle habits (cigarette smoking, alcohol drinking, medication use, nutritional supplementation and regular exercise). Associations between dietary intake and diseases (such as osteoarthritis, hyperuricemia and metabolic syndrome, diabetes, fatty liver diseases) were evaluated.  Ethical opinions:  After the assessment of the Ethics Committees on Research of the Xiangya Hospital, Central South University, the research is qualified for the safety and fairness, there will be no harm and risk to the study population. Participants are volunteered to be included in the research, written informed consent was obtained from all subjects in this study. Participants’ privacy will be protected during the study. No competing interests exist in the research. | | | |
| * Classification: 1= pathological tissues; 2=human trial; 3=human tissue or cell trial  4= embryo clone study; 5=other | | | |
